# Ellis’ Medical Formulary, Correction

**Published:** 1846-05-01

**Authors:** 


*Ellis's Medical Formulary. Correction.*—The Publishers of this Work respectfully request those persons who have the seventh edition, to correct a typographical error for the "MEDICATED HYDROCYANATE OF POTASSIUM," at page 83; wherein the symbol for an ounce is used in place of that for a drachm. The following is the correct prescription, and corresponds with the proportions directed in all the previous editions of the Work:

℞. Potassii hydrocyanici medicata, ʒj.

Aquæ destillatæ, Oj.

Sacchari purificati, ℥iss.

Fiat solutio.—Dose, a table-spoonful, night and morning.

Those of our readers who have the Work above referred to, will perceive at once the importance of making the correction specified.

